# Barriers and Considerations in the Design and Implementation of Digital Behavioral Interventions: Qualitative Analysis

**DOI:** 10.2196/34301

**Published:** 2022-03-30

**Authors:** Gabriela Marcu, Steven J Ondersma, Allison N Spiller, Brianna M Broderick, Reema Kadri, Lorraine R Buis

**Affiliations:** 1 School of Information University of Michigan Ann Arbor, MI United States; 2 Department of Obstetrics, Gynecology, & Reproductive Biology and the Division of Public Health Michigan State University East Lansing, MI United States; 3 Department of Family Medicine University of Michigan Ann Arbor, MI United States

**Keywords:** computers, mobile apps, screening, brief interventions, diagnosis, computer-assisted/methods, surveys and questionnaires, motivational interviewing, therapy, implementation, qualitative, mobile phone

## Abstract

**Background:**

Digital behavioral interventions have become increasingly popular for their ability to support patient diagnosis and treatment, chronic disease self-management, behavior change, and adherence to recommended care. However, digital intervention development is impeded by challenges such as limited technical skills, limited access to developers, and cost. The purpose of this study is to elicit in-depth qualitative feedback from intervention developers who have interest in digital behavioral interventions but lack programming skills regarding the barriers they experience and key considerations in the design and implementation of digital interventions.

**Objective:**

This study aims to understand barriers in the design and implementation of digital behavioral interventions, as well as to identify key considerations for researchers who are developing these interventions.

**Methods:**

We conducted semistructured qualitative interviews with 18 researchers who had experience either designing (but not coding) digital behavioral interventions or running research studies with them. Participants were a convenience sample of users of the Computerized Intervention Authoring System platform, an existing no-code development platform for building digital intervention content, and were recruited through either direct email solicitation or snowball sampling. All interviews were conducted and recorded over videoconference between February and April 2020. Recordings from interviews were transcribed and thematically analyzed by multiple coders.

**Results:**

Interviews were completed with 18 participants and lasted between 24 and 65 (mean 46.9, SD 11.3) minutes. Interviewees were predominantly female (17/18, 94%) and represented different job roles, ranging from researcher to project/study staff. Three key barriers in the development of digital behavior interventions were identified during interviews: lack of cross-disciplinary understanding; variability in recipients’ technology access, infrastructure, and literacy; and the idea that evidence-based in-person interactions do not translate directly to digital interactions. Interviewees identified several key considerations that interventionists learned to prioritize, which have the potential to overcome these barriers and lead to successful interventions.

**Conclusions:**

Barriers in the development of digital behavioral interventions are often created by a lack of cross-disciplinary understanding, which can lead to difficulties conceptualizing interventions, unrealistic expectations in terms of cost, and confusion about the development process. Moreover, concerns about research study participant characteristics and access to technology, as well as the translation of in-person interventions to digital, are apparent. Appropriate training in how to work with software development teams may help future digital behavior intervention creators overcome these barriers and may lead to new, exciting innovations in this space.

## Introduction

### Background

Digital behavioral interventions have become increasingly popular for their ability to support patient diagnosis and treatment, chronic disease self-management, behavior change and adherence to recommended care, and primary prevention [1]. They have demonstrated broad promise in terms of efficacy [2-8], but evidence for specific use cases tends to be mixed [9-11], with engagement and retention being particular challenges [9,12-14], as well as low-quality evidence [10,15,16].

There is tremendous room for innovation in this area. However, developing digital interventions is often out of reach for many research teams because of the challenges of developing relationships with software developers, the costs of custom software development, and the need for technical expertise, all of which serve as barriers to entry in this field. Critically, researchers need pilot data to access grant funding large enough to develop these types of interventions but may not be able to collect pilot data without at least a working prototype. Moreover, the constant need to update and adapt custom tools for other purposes means that research teams are constantly in the process of reinventing the wheel with each new intervention. Finally, most digital interventions are built using a particular technology stack by a particular group of developers, such that sharing or building on existing interventions is a significant challenge, even with the few apps that make their source code openly available.

No-code platforms, also known as authoring tools, may offer a solution to these challenges. Part of what has been called the no-code revolution, these platforms are designed to allow citizen developers [17] to build apps using simple graphical user interfaces. By providing templates and other structured step-by-step processes for building custom interventions, these platforms enable the creation of technology-delivered interventions without the need for computer programming or technical knowledge. For example, an increasing number of services now enable anyone to create their own website, including relatively complex functions such as e-commerce, responsive web design, and analytics. No-code platforms could similarly make the development of digital interventions faster, more accessible, and easier to edit and share by providing a framework within which researchers can build or tailor an intervention. A successful platform could be to the creation of digital behavioral interventions what Microsoft PowerPoint is to the creation of slides for a presentation: a tool facilitating an explosion of content generation.

In contrast, as with Microsoft PowerPoint, democratization of software development does not solve all challenges in the creation, testing, and implementation of digital behavioral interventions [17]. In this study, we sought to explore these challenges, grounded in the experiences of researchers who have played a range of roles with digital interventions. The purpose of this 2-part qualitative investigation is to better understand the motivations, needs, constraints, and experiences of researchers who have looked to digital interventions in their work addressing behavioral health.

### Objectives

In this paper (part 2), we seek to document common barriers reported by intervention developers, as well as key considerations, in the design and implementation of digital interventions. In a companion paper (part 1), we seek to document reasons why researchers who study behavioral health focus their efforts on digital interventions, as well as their perspectives on the perceived benefits that digital approaches afford researchers and their intervention recipients [18].

## Methods

### Overview and Ethics Approval

This study was conducted as part of a redesign of the Computerized Intervention Authoring System (CIAS) platform, a web-based digital behavioral intervention authoring tool for researchers. This study included previous and current CIAS users as well as CIAS-naïve users, all of whom had experience developing behavioral health interventions. We conducted semistructured interviews with these intervention creators to better understand their needs, perceived barriers to designing and implementing digital interventions, and design considerations for behavioral intervention development. COREQ (Consolidated Criteria for Reporting Qualitative Studies) guidelines were followed [19]. All methods used in this study were approved by the University of Michigan Human Subjects Review Board (HUM00171197) and Wayne State University (IRB-19-10-1340).

### CIAS Platform

CIAS is a Health Insurance Portability and Accountability Act–compliant no-code web app designed to allow users to easily build, edit, and share web-based interventions without coding or other technical expertise of any kind. CIAS gives interventionists the ability to develop tailored and personalized text-based interventions that can be narrated by an animated and emotive character capable of multiple different actions and voices (supporting >40 different languages, with male and female versions of most and a range of accents or dialects for some of the more commonly spoken languages). CIAS supports intervention building features such as multiple question types, natural language reflections, branching and tailoring, and integration of video content. Further details on CIAS can be found in the part 1 companion paper [18]. With support from the National Institutes of Health (EB028990), an all-new version of CIAS (3.0) is currently being developed as an open-source and noncommercial research resource. This research was conducted as part of the CIAS 3.0 redesign activities.

### Participant Recruitment

Participants were recruited via email solicitation from a convenience sample of CIAS users and researchers who have expressed interest in using CIAS, via snowball sampling, or via an email to a listserv of University of Michigan Department of Family Medicine staff members familiar with conducting digital interventions. To be eligible to participate, users were required to be aged at least 18 years.

### Study Procedures and Data Collection

All interviews were conducted in single sessions via teleconference between February and April 2020 by 2 trained research staff (BMB and ANS). Participants did not have prior experience with the interviewers, nor did they have access to interview guides prior but knew that they would be participating in redesign efforts. All participants provided verbal consent, and all interviews were recorded for later transcription. Interviewers also took field notes, which were available for later analysis. Interviews were conducted until saturation was reached. Transcripts were not returned to participants for review, nor were participants asked to provide feedback regarding the findings. All participants were offered US $20 for their participation, which was delivered in the form of a check via US mail.

### Analysis

To analyze the data, the 2 study team members (BMB and ANS) debriefed after each interview. During this debrief, individual notes were compared, which were gradually synthesized across interviews. Interview transcripts underwent 2 rounds of inductive thematic analysis, which was conducted by 2 coders (BMB and ANS); the first focused on identifying patterns, which later became themes, and then a second validated themes and confirmed connections between them.

## Results

### Overview

We invited 24 current and former CIAS users to participate, and 17 agreed (17/24, 71% response rate). In addition, we recruited 1 additional CIAS-naïve user who was familiar with digital health interventions from the University of Michigan Department of Family Medicine. For our 18 interviews, the average duration was between 24 and 65 (mean 46.9, SD 11.3) minutes. Participants were predominantly female (17/18, 94%) and represented different job roles ranging from researcher to project or study staff. Table 1 presents the aggregate interviewee characteristics, and Table 2 presents the brief descriptions of each interviewee.

**Table 1:** Aggregate interviewee characteristics (N=18).

Characteristics			Values, n (%)
**Gender**			
	Male	1 (6)	
	Female	17 (94)	
**Job title (all that apply)**			
	Researcher	13 (72)	
	Psychologist	4 (22)	
	Other (project manager or coordinator and research assistant or associate)	5 (28)	
**Employer**			
	Academic institution	15 (83)	
	Foundation	1 (6)	
	Contract research organization	1 (6)	
	Other (nonprofit research organization)	1 (6)	
**Race**			
	African American or Black	4 (22)	
	White	13 (72)	
	Prefer not to answer	1 (6)	
**Ethnicity**			
	Hispanic or Latino	2 (11)	
	Non-Hispanic or Non-Latino	16 (89)	
**Education**			
	Bachelor’s	6 (33)	
	Master’s	4 (22)	
	Beyond a master's degree	8 (44)	
**Self-reported proficiency with CIAS^a^ (n=17)**			
	Novice	3 (18)	
	Proficient	4 (24)	
	Advanced	7 (41)	
	Expert	3 (18)	

^a^CIAS: Computerized Intervention Authoring System.

**Table 2:** Individual interviewee descriptions.

ID	Self-reported title	Employer type	Highest level of education obtained	Gender
P1	Researcher; psychologist	Academic institution	Beyond a master’s degree	Female
P2	Researcher	Foundation	Master’s degree	Female
P3	Project manager	Academic institution	Bachelor’s degree	Female
P4	Psychologist	Academic institution	Beyond a master’s degree	Female
P5	Project manager	Academic institution	Master’s degree	Female
P6	Researcher	Academic institution	Beyond a master’s degree	Male
P7	Researcher	Academic institution	Beyond a master’s degree	Female
P8	Researcher; psychologist	Academic institution	Beyond a master’s degree	Female
P9	Researcher; psychologist	Academic institution	Beyond a master’s degree	Female
P10	Researcher	Academic institution	Bachelor’s degree	Female
P11	Researcher	Academic institution	Bachelor’s degree	Female
P12	Research assistant	Academic institution	Bachelor’s degree	Female
P13	Researcher	Contract research organization	Master’s degree	Female
P14	Researcher; research project coordinator	Academic institution	Master’s degree	Female
P15	Researcher	Nonprofit research organization	Beyond a master’s degree	Female
P16	Research associate	Academic institution	Bachelor’s degree	Female
P17	Researcher	Academic institution	Bachelor’s degree	Female
P18	Researcher	Academic institution	Beyond a master’s degree	Female

### Barriers in Designing and Implementing Digital Behavioral Interventions

Interviews revealed three primary barriers: lack of cross-disciplinary understanding; variability in recipients’ technology access, infrastructure, and literacy; and evidence-based in-person interactions do not translate directly to digital interactions.

#### Barrier 1: Lack of Cross-disciplinary Understanding

The first barrier commonly described by interviewees was the technical knowledge and resources required to use digital behavioral interventions. For some, this was the primary barrier: “I think the biggest barrier for many researchers is the actual building of the software itself, or how the intervention will be delivered” (P7). Building even a simple digital intervention requires the appropriate technical expertise and project management skills. Consequently, a significant amount of effort must often come from software developers who are outside the clinical research team, which increases the cost. The resources required were described by our interviewees as prohibitive, as well as difficult to anticipate:

The cost of actually developing an app is, I think, prohibitive for a lot of researchers....I think it depends also on your resources and the amount of time you have, you know, we have a small pilot grant from NIH, so we don’t have a lot of resources and we don’t have a lot of time and I think, I mean, I certainly didn’t know going in...it just seems like everything, every piece of this, takes longer than we anticipated and the programming is a lot more intricate.

P15

The bounds of a clinical team’s technical knowledge may limit their ability to not only implement their ideas through technology but also to even envision what possibilities are available to them:

I think it’s just a lack of knowledge around technology. Like, what options are available. I think that the go-to thought process when people think digital interventions is telehealth...but there’s a lot more to that. So, I think just knowing that there are other options is a barrier. A lot of people are limited in that. But then once they realize that there are other options...it’s accessibility to these things. Like, what is out there? How much does it cost? How do I do it? You know, a lot of the people designing these interventions aren’t…they don’t have a background in technology.

P16

Conversely, those with technical knowledge who develop tools are often limited in their understanding of how interventions should be delivered. Interviewees discussed the challenges of using digital interventions that had been created without input from clinical experts:

The people who are developing these interventions are not people who deliver them. So that’s a problem, I think, because sometimes it’s really hard to envision what should it really look like in the development stage when you’re not the kind of person who would be sitting across the table from someone, right? So like, you can’t figure out what the gap is because how would you know? Because you’ve never been sitting across the table from someone.

P1

As a result, interviewees found that existing software programs had significant limitations in how they could be used for developing and delivering interventions. For example, they did not allow the interviewee to edit and control important aspects of an intervention: “I think the lack of editability of the program, I think that’s the biggest thing that’s frustrating for me” (P12). Interviewees therefore indicated the need to truly combine knowledge of behavioral interventions with knowledge of technology that can deliver them. However, those who had worked closely with software developers noted the challenges inherent in this cross-disciplinary work:

That can be a barrier because oftentimes there’s disciplinary differences that challenge that experience, you know, speaking different language, computer programmers not quite “getting” behavioral intervention and the needs of the behavioral interventionist.

P7

This interviewee also went on to explain that the level of investment in technology involves trade-offs that are commonly discussed and debated among researchers:

Particularly when we’re at conferences and seeing the work of other people, is that process of working with software engineers and developing software from the ground up is not a short process...I’ve seen animated programs that are really fun and interactive and interesting, but they also took 2 years to build...So, we’ve kind of talked about that trade-off before. Then you think about the fact that most people stop using an app or web-based type of a program, typically in a short period of time. You’ve got that huge investment in that fancy intervention software and then people are using it for a short period of time and never looking at it again, and that’s a huge investment of resources for what could be a limited return and an unknown efficacy.

P7

The level of resources required to develop a digital intervention is a large investment and one that comes with some risk. Therefore, setting realistic expectations for timeline, cost, capabilities, and the return on investment is necessary before turning to digital interventions. One interviewee explained how they share their past experience to help other researchers adjust their expectations:

So, if they say “oh, we want a long course [for the intervention], we have multiple interventions, we have $5,000” something like that. I will just tell them that $5,000 is not enough, we need a lot more than that, however, no matter what you have, you can usually get something, but it may not be what you want. So, it’s $5,000—it’s going to be crude messaging, it’s going to be texts, it’s going to be tailored audio tracks, or mailed—simple feedback. You could do that, but you’re not going to do anything fancy with video, or anything like that, for a couple thousand dollars. I guess that’s the last thing, that’s helping people to budget what kind of money they have, what kind of expectations are.

P6

Planning and budgeting an intervention with realistic expectations can also help interventionists to think through how presentation of the content will affect intervention efficacy. Some interviewees shared their concern that the look of the digital intervention is linked to its credibility and perceived value: “Better optics might make participants take it more seriously” (P12). Interviewees therefore felt that investing in the aesthetics of the intervention could even impact engagement and efficacy.

Finally, we note that interviewees mentioned a set of technical barriers after the software development had been largely completed. As interventionists finalized, pilot-tested, and prepared an intervention for recipients, adjustments and edits still needed to be done within the code. For example, changing the terminology used on a button may seem straightforward; however, because the label on the button is hard coded into its functionality, the interventionists will not be able to change it themselves. The process of communicating every single change, large or small, and waiting for all of them to be completed can significantly extend project timelines. One interviewee described this extensive process and how experiencing it changed the way they would approach writing grants by accounting for each step in the process:

The communication [with the software development company] and the ability to sort of get something corrected just adds time to it relative to things that we could otherwise have done in-house....We would submit what we needed. They came back with things they could do easily, things that would take more time, things that would cost more money. And so that was probably several months of just sort of figuring out what was going to be feasible given the limitations of grant funding. And then probably another couple of months of back and forth finalizing items and then the student pilot tested with a couple of participants and worked out a few more glitches before we launched...But that whole process, you know again not having really done that with another company...just made me appreciate kind of, if I had to write another grant with that company how I might do it.

P9

Digital behavioral interventions require significant knowledge from multiple disciplines, which naturally raises costs and increases the complexity of project communication and timelines. Through experience with digital interventions, interventionists get better at envisioning what they can do with technology, having appropriate expectations for a software development project, and managing costs.

#### Barrier 2: Variability in Recipients’ Technology Access, Infrastructure, and Literacy

Technical barriers from the recipients’ side include access to technologies such as mobile devices or Wi-Fi, literacy with these technologies, and the availability and cost of local infrastructure such as internet connection and data plans. These factors are often underestimated when digital interventions are being designed and planned and also lead to greater resources that need to be contributed by interventionists, such as supplying Wi-Fi, data plans, or additional technical support. In addition, as operating systems (eg, Android [Google] and iOS [Apple Inc]) are updated over time, digital interventions may require recipients to install these updates on devices to ensure their continued operation.

Interviewees referenced the digital divide, the significant disparity between those who have access to technology and have fluency with technology and those who do not. Most commonly, interviewees mentioned mobile devices such as tablets (eg, iPads) and smartphones, as these are the primary technologies currently used for delivering behavioral interventions:

Not everybody has a computer. Not everybody has an iPad at home. Most—a lot of people do have smartphones, but even then, like it doesn’t mean that they’re going to be able to access the information or they’re willing to access the information.

P2

In order for technology to effectively facilitate a behavioral intervention, recipients must have not only access to the right device but also the appropriate technology literacy to operate and troubleshoot the device. Interviewees experienced the digital divide between themselves and the people they were trying to reach, acknowledging that the decision to use a digital intervention was a much easier one based on their own considerations compared with those for recipients:

For us, it was an easy idea for us to, you know, be like “yea, we’ll do it on an iPad! No big deal.” But then, in talking to the mothers that we’re potentially going to be using this behavioral intervention with, we quickly realized that a lot of these participants have probably never held an iPad in their hands before and that kind of totally changed the way we need to develop our intervention to where it’s, not only are you creating your intervention, but you have to create the instructions and infrastructure in place so that they can even experience the intervention in the way that you think it’s going to go.

P13

In ensuring the right experience for intervention recipients, this interviewee alluded to the instructions needing to match their technology literacy, a consideration very similar to health literacy. With regard to infrastructure, this can include the type of internet connection accessible to a recipient in their neighborhood, as well as the data plan they have on their phones:

With our population...I know everyone has a phone but a lot of [the] women use prepaid phones with minutes. And they have...a limit on how much data they can use.

P16

Often, the varied technological constraints can be underestimated and may not be considered when digital interventions are chosen, designed, and initially implemented:

The extent of it came up organically, like just how much we kind of took for granted when it came to technological literacy with these [participants]. I think we didn’t realize that we would have to, in some cases, provide our own Wi-Fi, and that we can’t just ask these [participants] for an email address to get their feedback report, so things like that came up organically, the whole idea that we needed to know where these people were at with technology.

P13

Interventionists also did not always consider compatibility issues based on which versions of hardware or software recipients were used. For example, an older tablet may cause more issues or not be able to run the intervention. As operating systems evolve with updates regularly released, users must download and install these updates on their devices; otherwise, the intervention may no longer run. When an intervention stops running as expected, the cause is not always obvious to the user, and they may need help troubleshooting. Although these challenges are par for the course with any software project, interventionists may not be prepared for them:

All of a sudden, we’re dealing with hot spots, and intermittent access, and old computers, and updates. The versions, what we do now, Android will update a version and all of a sudden it will break, it won’t work. So, our people in the field were like, “oh, it’s not working, people are sitting around, the clients are getting frustrated,” and your tech people will say “we don’t know what’s happening, it works fine on our—,” you know. Computers are way more difficult, so those are some of the reasons people don’t do it is because they don’t know how.

P6

Interviewees had learned how much extra work was required from their research team when technical challenges arose for the recipients:

For this new clinical trial, when they’re going to be doing it by themselves, like it’s already hard enough to get people to enroll in these studies and then for it to not work properly. And then I’m, they’re going to reach out to me, my project manager, like, “well, this is not working.” And then it makes more work for them, okay, go take a screenshot of this point where it didn’t work and then email it to me. It’s like, oh it’s a whole production.

P12

Interviewees were therefore concerned about the effects on both the recipients and research staff. Moreover, technical difficulties with a digital intervention could stand in the way of effective behavior change, jeopardizing impact on the recipients, as well as the course of research:

I’m a little bit concerned about potential technical difficulties that they might experience that might lead to the intervention not being completed, which would then limit our ability to test the efficacy of the intervention.

P7

Designing an intervention that meaningfully engages a recipient in the process of behavior change is challenging enough on its own, but a digital intervention adds multiple levels of technical considerations.

#### Barrier 3: Evidence-Based In-Person Interactions Do Not Translate Directly to Digital Interactions

The final barrier we found concerns the shift interventionists had to make from designing an in-person intervention to designing a digital intervention. Interventionists look to technology for many perceived advantages [18]; however, interviewees pointed to the difficulty of adapting person-delivered interventions to digital forms. For example, despite the capability to program a digital narrator in CIAS so that it can adjust content in reaction to input from the user, interventionists still missed the richness of other cues they were used to relying on during in-person interactions:

Trying to translate the patient-provider interaction and the types of feedback and information that goes into that—how can you program that? [The digital narrator] is pretty much 100% scripted, he doesn’t really do a lot of responding to cues.

P7

The CIAS platform allows interventions to be delivered through a digital narrator whose voice reads out the intervention text and whose physical form appears in the interface as a human or animal. In this physical form, the digital narrator can be animated for a range of actions, such as talking, smiling, reading, waving, and pointing to items within the interface. The narrator can also ask recipients for their preferences, reactions, and thoughts at any point and can provide verbal reflections of that input (eg, “It sounds like this has been pretty hard for you”) and branch to different areas of content based on that input. However, interventions built using the CIAS platform cannot read facial expressions or body language and cannot interpret unscripted input from recipients, leading to challenges in forecasting or collecting a range of likely reactions or preferences so they can be included in the intervention:

I think part of the challenge that we’ve had is we’re often trying to take what we would do in a face-to-face and think about how that would translate to a computerized delivery, which there’s obviously going to be elements of that that are lost with the computer delivery. [The narrator] can squawk and flap his wings and make cute expressions, but he can’t really interpret the non-verbal patient sitting across from him so to speak...When you’re in the face-to-face setting, you obviously can use non-verbal and other types of communication to inform your thinking: looking at patients’ records, looking at other sources of information, downloading meters, and other things that can be conversation start points that [the narrator], he can’t necessarily do.

P7

Although digital interventions built with CIAS allow for personalization, tailored feedback, and empathic reflections, the need for a priori entry of possible preferences and reactions is a departure from the flexibility and improvisation that is possible with person-delivered interventions:

I guess the biggest problem that I would say is that, you know, you’re just in a forced choice kind of framework...So like, for example...if I were to ask you “hey, what do you think would...what would help you supervise your kid every day?” So, let’s say you...rate yourself as motivated but low on self-efficacy...so, if I’m sitting across the table from you, I can just say to you, “well, what are some things you’ve tried?” or “what do you think would work?” and we can have an open-ended conversation. Whereas in the program, as the developer, I have to presuppose what your likely barriers are, and put them in there, so you can click some boxes.

P1

Interventionists, therefore, experienced greater difficulty when they approached the design of a digital intervention in the same way as a traditional human-to-human intervention. Some described the need instead for computational thinking or thinking of an intervention based on the characteristics and advantages of computers, such as logic and chunking:

But the difficult thing is to bridge that gap between what can be done in person versus what can be done online. What helps me is to think through sort of logical rules or how would you chunk things. And there are an infinite number of possibilities of what could happen in an in-person interaction.

P6

However, as interventionists drew on behavioral theory and approaches, such as motivational interviewing (MI) [20], their expertise in applying them was primarily via human interaction, making it difficult to envision a different type of intervention. For example, interviewees pointed out that the ability to incorporate recipient responses into the course of the intervention was a key component of MI:

The most challenging thing is taking the human part out and trying to put that into mobile platforms...so, for example, like I said with MI, branching out the reflections and making it so that you could take someone’s motivation and put it on a scale...that was challenging versus asking someone “hey, can I know more about this?”...I think that’s a challenge...because as a human being, you could just adapt to [their response].

P5

The loss of nuanced reactions they were used to observing in person also meant fewer opportunities for gathering rich qualitative data:

I think, with the regular behavioral interventions that I work on it’s a lot more communication with participants and like face-to-face interaction which is kind of nice because you’re getting in like real-time reactions and kind of some good qualitative data. But I guess with [the digital intervention] they’re doing on their own you’re not kind of seeing their reaction to the actual intervention.

P3

Human contact is, of course, not easily replicated with technology, which some interviewees saw as a significant loss:

So, the biggest thing that I would consider is, a lot of participants that I’ve seen in the past, the number one thing that they get out of being involved in research is that they get to talk to somebody, they get that communication, they get that personal connection. So, like if there’s a chance that they’re going to—in their eyes—lose that, just the way that you approach how you’re going to transition it. Like, we’re not going to be losing contact, we’re going to still be in contact, and how you’re going to be in contact. Because a lot of people that we deal with, they have severe depression and anxiety and so they participate in these things obviously to try and you know, reduce those symptoms. But also, because they don’t have anybody at home so they like seeing somebody in person.

P11

This interviewee highlighted the potential benefits to intervention recipients, especially when working with sensitive topics and vulnerable populations, as a key consideration when choosing a digital intervention. Combining digital intervention with strategically positioned human touchpoints may also be an effective way to maintain such benefits for recipients.

However, integrating digital interventions smoothly into the experience and constraints of a recipient or an interventionist may benefit from new approaches. When designing a digital intervention, we found that interviewees often work to translate traditional interventions directly, maintaining a very similar model or approach that they had previously used in person. For example, one intervention relied on an avatar, whose role was viewed similarly to that of a human counselor helping the recipient to navigate the steps of the intervention:

We have a little [avatar of a] woman that walks you through the whole process. She kind of acts as the counselor for the participant and talks them through everything, and we build scripts for her. Kind of, build like, questions and answers and get to know the participant through CIAS.

P12

This interviewee discussed the process of writing a script for the avatar, much like a script might be generated to support consistent human delivery of an intervention. However, achieving a flow similar to that of a natural conversation was a challenge with this approach:

I think at times it tends to, and this might be on the side of the script building and our side of putting CIAS together in the back end, but I’ve noticed at times that it tends to lag or, kind of lack the flow, it needs to kind of seem like a conversation.

P12

Digital interventions need not be designed according to the traditions and best practices that have been developed with face-to-face interventions. Instead, digital interventions may be improved by envisioning new paradigms.

### Key Considerations for Designing and Implementing Digital Behavioral Interventions

#### Overview

As interviewees shared the barriers and lessons learned from past experiences with various interventions and recipients, we identified three high-level considerations for designing and implementing digital behavioral interventions: understanding the population and context, integrating the intervention within ecologies of care, and technical staffing and preparation. A strong focus on these considerations can help address the barriers we have described previously.

#### Understanding the Population and Context

As discussed earlier, the process of adapting behavioral approaches to digital interactions with recipients is one of the greatest barriers to effective implementation of digital interventions. Therefore, the first step in understanding the technological context of recipients is critical not only for ensuring appropriate access and literacy to enable intervention delivery but also for envisioning how each step of the intervention will be experienced by recipients, so that its delivery is evidence based and recipient centered. Interviewees gained this understanding through qualitative methods with the population and frontline interventionists who interact directly with the population (eg, via home visits):

In our first focus groups, we were talking to both home visitors and moms, [asking]: How comfortable are you using technology? If someone handed you an iPad and said “go through this program on your own,” how comfortable would you be doing that? What are some things that would get in the way of you being able to do that? And then asking the home visitors, “when you go into a home, is there Wi-Fi? Because this is a web-based program.” Things like that. And then we had them sit down and go through the program and analyze both their experience with it, just as the platform in general, and then also nitty-gritty of the wording of the content and what we’re trying to accomplish.

P13

This interviewee highlighted some of the best practices in designing digital interventions: starting the process by engaging with the population through a method such as focus groups to gather information pertinent to their experience and assessing each aspect of the intervention through their actual perspective, from the experience of interacting with the technology to clarity of the content presented. Another best practice mentioned was pilot testing, also known as usability testing, which examines how the recipient interacts with the intervention to identify how that experience might be improved:

We plan to do a small, pilot trial to test it out in home visiting, and because it’s a pilot we consider [it] part of the design process where we’re really looking at feasibility and acceptability, which parts work and which don’t, and then we’ll continue to tweak it from there.

P15

As this interviewee points out, there must be an iterative process of testing and revising. As such, obtaining input on aspects of the design from end users should occur from the early stages of designing a digital intervention. Deeper engagement, known as participatory or community based, can involve the population in not only evaluating the intervention but also contributing ideas to its design:

I think everyone needs to do more of a community-based approach, if they can, to where they’re involving the target population in the creation of [the digital intervention].

P13

Engaging the population in evaluation, or even design, can help address a range of concerns about how the intervention will be received. For example, interviewees mentioned that when it comes to the overall aesthetic of the intervention, “the way it looks is how seriously someone is going to take your research regardless of the content” (P14). Interviewees also made comparisons to the aesthetics of common mobile apps, such as social media apps. Referring to a prior version of CIAS, one interviewee said:

It could just look a little bit more, you know, like the social media apps that everybody uses all the time. And like if we saw a social media app that looked like that, you’d be like “ew, what is this app, like it looks weird.” I love CIAS, not to say that I hate it, I appreciate it, but I’d like a graphic redesign of CIAS to make it a little bit more appealing to the eye.

P12

Such natural comparisons are important to consider because how an app looks and feels to a user and how the intervention content is presented can be linked to perceived value or trustworthiness of the intervention as well as the research study.

Finally, digital interventions expand the possible contexts in which recipients can be reached, changing the way they must be designed. Deciding on the geographical constraints for reaching a certain population has important implications for the intervention, and interviewees described this decision as a balance between convenience for the recipients and convenience for the research team:

Do you want to restrict it to certain geographical areas...what’s your population?...Where are these people located, the time zones? Because we have people like in California. We have to consider you know, because we have to do follow up calls every six weeks after they’ve completed their treatment and sometimes we have to consider that they’re three hours behind us, so what time might be good for them, we have to accommodate, because they are doing us a favor by participating in our study so if that means that we have to call them at 8:00 pm our time that’s fine....So those are the kind of barriers, it just really depends on how widespread you want your remote study to be.

P11

Interventionists took into consideration a range of factors about the population they wanted to reach with a digital intervention, working to understand their background, technological context, how they would interact with the digital intervention, what their expectations may be of interactive technologies generally, and how geographically distributed they may be.

#### Integrating the Intervention Within Ecologies of Care

Interventionists also considered how an intervention might be integrated within ecologies of care—existing services and supports, both in virtual and real-life contexts. Our interviews indicated that researchers have a range of ideas for integration but have been constrained in achieving all of the features they envision. For example, they thought recipients should be able to schedule a health appointment and even see a clinician at a distance using the same or another platform:

Having a feature that if a client wants to have a digital health appointment for something, they could set that up on their own in the [digital intervention]. They’re scheduling it and it’s happening on the platform.

P16

Another potential feature mentioned was supporting patients to monitor their progress toward goals, which can include reminders, strategically timed prompts to action, or visual feedback on what it will take to meet their goals. One interviewee described the use of this approach for long-term engagement:

Have your [digital interventions] just send them text messages: “hey you set a goal for whatever, just make sure you do that today.” That’s the cheapest and easiest way [to improve patient compliance]. If you can tie your [digital interventions] in [with] some long-term prodding of your patients in the next week or two or three or whatever, in a way that clinicians think they’re going to reap that. If the patient has been prompted to do something, the clinician thinks: “gee, the next time they come in, maybe they’ll have gotten their material together and put it in the binder and developed a diet, talked to a nutritionist, or visited a grocery store, or found out more about where they can get nicotine replacement therapy.” So all those I think are selling points.

P6

Similarly, care transitions, such as leaving residential or inpatient care, are a great challenge in which technologies could provide support. Interviewees described roles for digital interventions across multiple touchpoints in one’s journey with treatment for substance use:

I think I could see [digital interventions] particularly serving a role in transitions from things like residential to outpatient treatment. That’s that window I think we’re all still really struggling with is, how do you get [recipients] to maintain abstinence when they leave residential care? Where I think trying to build things in with brief interventions would be a great place. So, I think within the addiction treatment realm of specialized services I would see [digital interventions] as something that could help with engagement at the front end of things—to provide some early counseling in a way that facilitates retention in treatment and even enrollment in some cases. And then I think in the longer term [digital interventions] could be used for interventions that help to reduce relapse risk and facilitate uses of care.

P9

Our interview findings suggest that even if the technology alone cannot provide meaningful interaction, the way it is integrated into other touchpoints of the research study itself can facilitate human contact that may be able to make a greater impact:

I don’t think [the digital aspect of the intervention] detracts from our interaction because we still have to get to know each other over the year that they’re in the study, so talking with them over the phone, and asking them really personal questions like adverse events form and things like that about their lives.

P12

Conversely, the way the technology is used can help minimize human interaction for the purpose of protecting privacy when engaging recipients on sensitive topics:

We also use headphones. So that’s something that we do to make sure that it’s anonymous, because the whole idea is that people don’t want to talk about these issues with their home visitors. So we’re giving them an iPad so that it’s just them and the iPad.

P2

When choosing a digital intervention, it is important to remember that recipients are not likely to interact only with the technology. Integrating the digital intervention effectively within the structure of the research study, as well as the broader landscape of services and supports, will enable a good experience for recipients and may even improve impact or outcomes.

#### Technical Staffing and Preparation

Finalizing, testing, and preparing a digital intervention to ensure that it is ready for use is a complex process with various considerations. Our interviews revealed the extent to which research teams need to have staff available to focus on each aspect of this preparation. For example, once scripts for the avatar were written, multiple members of the research team listened to how they would be delivered by the automated voice to ensure a good experience for the recipients:

[The PI] did a lot of the scripting, like, actually what the character would say and then she and I both would put it in there and listen to it and see how it flows...I think that we really worked hard to make the language understandable and modern. We didn’t use a lot of very complicated words. It is an automated voice, so we really played around with like how words sound.

P16

The flow of audio and visual features also needed to be reviewed on the same device and in the same way that recipients would experience them. CIAS enables interventionists to design their intervention on a laptop or desktop computer while previewing what the intervention will look like (eg, on the smaller screen of a mobile device). However, such previews will not convey the full user experience, for example, leaving out touch screen interactions, which will affect how intuitively and efficiently a recipient is able to navigate through the intervention. Therefore, researchers would ensure to review the intervention on the actual device their recipients would be using:

And then I think the last step is just making sure that it looks nice on the iPad. So, just going through it as many times and listening to it. … If I’m previewing [the intervention] on my laptop, it’s not going to be the same on an iPad.

P2

Interviewees also noted that a digital intervention may not look or perform the same across different platforms, such as tablets versus smartphones or Android versus Apple. These potential differences had implications for testing across platforms, as well as members of the research team having enough familiarity with operating different platforms so that they can troubleshoot during a study:

I have really tailored our intervention to a tablet screen. I haven’t looked at it on a phone or you know...on a computer in a long time. So, I like the cross platform capability to be able to like take something that’s working on one and make sure it’s going to look the same or be usable on another device—something I’m actively trying to look at right now.

P16

I have never used any digital intervention on an Android phone, so I’m not that comfortable [troubleshooting potential problems for recipients]. I would assume that it works the same way, but I don’t know. Or just things like, [the recipient’s] device functionality not being the same as ours [Apple device].

P12

In addition to testing the intervention on an appropriate device, interventionists needed to test it on an internet connection within the community that was similar to how the recipients would be accessing the intervention. This was especially important in communities or contexts in which internet connections may be unreliable or restricted (eg, behind log-ins, paywalls, or firewalls):

I’m going to ask my [research assistants] to go out into the community, whether that’s a library or to a Starbucks, or wherever and see if they can run through the intervention without getting kicked out.

P7

One interviewee worked as a project manager and spent a significant amount of time testing all branches of the intervention logic. As she suggests, CIAS or other types of platforms could potentially provide support specifically for this type of testing:

Where I have the most experience was doing the beta testing before we could actually run it...you had to test every single branch because there is no way within CIAS to like click a button to see the flow.

P5

Testing did not end once a study had begun. Research teams would have to ensure an adequate internet connection for individual recipients, who may be distributed across different contexts. This activity was so critical that it had been formalized as a part of study protocols:

In our protocol, we have outlined different ways to kind of go about that barrier [of having Wi-Fi]. Our participants can be all over [the state] or all over the US. They could be in places where there is just not Wi-Fi. So, our first thing is suggesting they go to a public place that has free Wi-Fi and we can help them figure that out. Is that a bookstore, or is that a little coffee shop or something, and then if they can’t, then we’ll direct them to our IT person and possibly work with them on getting them a mobile hotspot.

P11

If Wi-Fi could not be guaranteed, some study teams carried data cards in the field, that is, prepaid cards that can be inserted into a mobile device for internet connectivity without Wi-Fi. The data cards were used for either a recipient’s device if it did not have a data plan that could support the intervention or the research team’s device when it was more cost-effective than purchasing a data plan. The data cards added to the research assistants’ responsibilities for materials and troubleshooting:

So we have the data cards and we have the iPad for the site study I have actually been doing the digital intervention with. The upcoming one will be [on] their device, but still I would bring the [data] card and they would still use our iPad to do questionnaires.

P12

The approach of providing technology for recipients was a common way to overcome the digital divide and ensure a more consistent experience for recipients. This strategy still requires sufficient staffing on the research team to provide technical support, but troubleshooting becomes significantly more straightforward. For example, one research team distributed Kindle e-readers to recipients:

We knew that technology was going to be a downfall so we have a tech personnel on call to answer any questions that need to be....They are receiving a Kindle, so if they don’t have a computer, they don’t have a phone, it’s okay, they have the Kindle, and they get to keep the Kindle. We modify it so that it’s very easy, so on the home screen it’s like this is your app.

P11

Compared with asking recipients to use their own devices, supplying devices eliminates the need to provide instructions or support for downloading because the research team can ensure that study devices reach recipients with the app already installed on them. Some research teams also allow recipients to keep study devices, either as an added incentive or to avoid the challenging logistics of collecting the devices and reusing them at the end of the study.

Many digital interventions require recipients to have email addresses so that they can set up profiles or accounts. However, research teams cannot assume that all recipients would already have their own email address, and interviewees needed to plan for research assistants to take the additional step of helping to create one when needed:

If [the recipients] don’t have an email address, my research assistant works with them and she helps them create an email address just for them.

P11

Although support for creating an email address is easy to provide, this illustrates the need to be nimble and prepared to troubleshoot and assist, which can require a significant amount of time from research staff.

Finally, interviewees described taking the time to set recipient expectations for how they will experience the intervention. This activity was described not as a training but as an orientation or a demo, indicating that the technology itself should be intuitive to use, but recipients still ought to know what to expect as with any research activity:

We’re gonna talk with the participant during what we’re calling “intervention orientation,” where we kind of explain kind of how the intervention is going to be delivered and how it’s going to work.

P7

We’ve demoed it on a computer or on an iPad through a projector so that way people can see what it looks like. I’ve also gone in and created a short video...of what people can expect to see throughout the intervention, because I think it’s important for people when they’re receiving it to know what they can expect. So like, “this is what questions will look like.”...“this is what a link will look like, and this is what will happen if you click on a link.”

P2

After a research team has put considerable thought into designing their intervention and invested in the complex process of building the intervention according to their design, the key phase of ensuring that their intervention will be implemented effectively with recipients remains.

## Discussion

### Principal Findings

Our findings from these researchers with experience in digital intervention development are 2-fold. First, we isolated three common barriers: lack of cross-disciplinary understanding; variability in recipients’ technology access, infrastructure, and literacy; and evidence-based in-person interactions do not translate directly to digital interactions.

Second, we identified three key considerations that interventionists had learned to prioritize across different types of interventions and projects: understanding the population and context, integrating the intervention within ecologies of care, and technical staffing and preparation. These considerations are important from the outset of project planning and budgeting, and focusing on them can help address the barriers. We revisit each barrier to contextualize these findings and build on what interviewees had offered as their key considerations to using digital interventions, by adding our own recommendations.

#### Barriers in Designing and Implementing Digital Behavioral Interventions

##### Lack of Cross-disciplinary Understanding

Although digital behavioral interventions are often developed by multidisciplinary teams, the nascent ideas for these programs tend to start with either clinicians or researchers who do not have experience with software development or with developers with technical backgrounds but no clinical experience. As our interviewees were interventionists and not technical developers, themes that arose from this study centered on the lack of cross-disciplinary understanding that results when nontechnical interventionists seek to develop digital behavioral interventions.

##### Conceptualizing Digital Behavioral Interventions

Interviewees expressed concern about the lack of technical expertise on the part of intervention creators and the effects that this may have on interventions. The ideas for digital behavioral interventions conceptualized by clinical researchers are often built on the shoulders of other interventions. For example, a researcher may see an interesting intervention that applies to 1 disease state and brainstorm ways in which it could be adapted or replicated for use in another disease state. Although this approach is common, when it is used by nontechnical clinical researchers, the new idea may unnecessarily pigeonhole new interventions by ignoring different intervention delivery modalities (eg, websites, native mobile apps, web apps, and smartphone apps), design features (eg, manual self-monitoring and pairing with peripheral devices for objective monitoring), mechanisms for communicating content (eg, text, audio, video, and animation), or design decisions that the creator may not have considered or even be aware of.

##### Misaligned Expectations of Development Costs

Emerging technologies often remain out of reach for many interventionists because of their cost, which depending on factors such as complexity, novelty, and timeline, may vary greatly. Therefore, it is crucial for interventionists to align their expectations of what they want and need to develop, with the resources available to support intervention development. Misalignment with wants or needs and available resources often stem from a lack of experience with digital behavioral intervention development or the costs necessary to run and maintain an intervention, such as expenses related to hosting, security, bug fixes, and other maintenance costs. Most behavioral interventions used within research need to be implemented reliably at a scale within certain budgetary constraints. Interviewees voiced many concerns related to development costs, indicating that funds available for a given intervention development project were often far less than what was actually needed. Indeed, budget caps on federal and private research caps are often incompatible with actual market costs for high-quality software development and maintenance, which may stifle innovation. In particular, development costs are often greatest for new interventions, which are least likely to be competitive for large grant awards. Interventionists interested in advancing the state of the art in digital interventions often need to partner with technology researchers on more sophisticated and innovative interventions, often at a smaller scale, greater cost, and limited immediate ability to demonstrate impact on behavior.

##### Working With Software Developers

Interventionists often lack the knowledge and skills required to build digital interventions themselves and must partner with software development companies to create apps; however, doing so requires preparation that most researchers lack, at least initially. For example, even the process of identifying the right software development company can be a challenge. Some clinical researchers expressed confusion around their options when it came to hiring developers, saying that they did not understand what they should look for or how to know who to hire. In addition, before an intervention can be developed, the clinical researcher must have a clear vision and proposal for the intervention, including full details of each question, how it should be presented digitally, and an outline of all functionality. From hardware to software to the user interface, there are intricate details to a digital intervention that are time-consuming to design and will affect the quality of the intervention. Many of these details require careful decisions that researchers have not previously considered. Interviewees described lack of knowledge about what was even possible. Finally, developing and testing the intervention takes time and iteration. The processes, steps, and timelines imposed by working with a software development company can be quite lengthy and costly, growing more as researchers need even small changes made to a developed intervention. Working with outside development companies can create a disciplinary gap because those building the intervention are not typically knowledgeable about interventions. This can lead to more back-and-forth between clinical researchers and developers to be on the same page.

##### Understanding the Product Design Cycle and Development Timeline

Clinical researchers who do not have first-hand experience with the product design cycle used within software development may not know how to plan their projects around it. If they do not have realistic expectations, they can then find themselves with timeline issues such as delays and budget problems associated with not locking in functional requirements and specifications in a timely manner, difficulties in making system changes because of an advanced stage of coding, or failure to include necessary components or design processes into contractual language. These can all result in digital health tools that miss the mark both for the creators who envisioned the interventions and for end users. Moreover, many digital behavioral intervention projects are behind schedule from the moment they are funded because of unanticipated contracting delays. Ensuring adequate communication with the clinical researcher, who in this case is the client for which the software is being developed, is not typically the role of a software developer, programmer, or engineer. More commonly, the responsibility for understanding the needs of the client and end user of the software lies with consultants, product managers, or user experience researchers. These types of roles specialize in overcoming cross-disciplinary communication challenges and translating between technical language and the language of, in this case, the behavioral interventionist. Unfortunately, our interviews suggest that the prohibitive costs of software development may lead researchers to work directly with those who will write the code, asking them to take on a broad range of roles that are critical to a successful software project. Working with an experienced third-party vendor, or investing in these other roles and specializations in-house, can make cross-disciplinary collaboration more effective for creating an impactful intervention, and even thoughtfully target the most difficult problems, such as engagement with technology over time.

##### Meeting Recipient Expectations for Digital Technology

Interviewees shared their concerns about ensuring that their intervention aligned with expectations that their recipients have for digital technologies they use in their everyday lives. Given the ubiquity of high-quality free apps and mobile websites and the technology industry’s investment in the user experience of their products, consumers have high expectations for the appearance and functionality of interventions. The most prominent apps in their lives (eg, for social media, e-commerce, email, and search) are created by companies that invest considerably in the user experience, with entire departments dedicated to evaluating and continually improving the experience for their users. Apps that do not provide an experience that users have come to expect can generate disappointment, lower engagement, or abandonment.

The concerns of our interviewees echo the problem of engagement, which is well established in the literature [9,12-14,21,22] and which may be exacerbated when digital health tools do not provide a high-quality user experience. Interventionists should therefore draw from industry best practices to meet their recipients’ expectations. Asking end users of a digital intervention to interact with it, and assessing their experience, is an essential best practice [23] for ensuring the technology is user-centered and intuitive to use. For example, mockups of how the digital intervention will look can be shown in a study by Melles et al [23] or as a static image on a tablet. Users can then be asked to think aloud [24] as they review the information presented, react to the information, and describe what actions they believe they can take next. Such qualitative methods are the most effective [23] to ensure that the digital intervention is designed in a way that matches the mental model of the population. Achieving an adequate user experience can be costly, not necessarily because this requires complexity (in fact, current technology trends favor minimalism), but by investing in a user experience designer or interaction designer who will know how to meet user needs and expectations, including up-to-date standards and conventions to make it feel modern and credible. Moreover, engaging with the population as various aspects of the intervention are being designed can reveal how different design choices will affect the way they experience the intervention, providing opportunities to correct the course as needed while the intervention is still taking shape. These activities add further complexity to the timelines and budgets of software development projects we previously discussed. However, these activities are commonly adapted to fit the constraints of each project and can even save time and money when used early and strategically.

#### Variability in Recipients’ Technology Access, Infrastructure, and Literacy

Interviewees in this study expressed many concerns about the technology access, infrastructure, and literacy of target end users, and these issues need to be considered by digital behavioral health interventionists, particularly those that work with underserved or marginalized populations. Although access to computers and smartphones may be high, access is not ubiquitous in all populations. Moreover, technology infrastructure is not equitable across the country, particularly in rural and underresourced urban settings where access to affordable, dependable, high-quality, high-speed broadband may not be readily available for all [25,26]. Furthermore, intervention-generated inequalities may arise where already advantaged populations gain greater advantage through digital interventions than do those who are less advantaged [27].

Finally, technology literacy is required to ensure that a device is connected to the internet, any peripheral devices such as a keyboard or wearable technology are paired, effective security measures such as passwords are used, and software updates are downloaded and installed in a timely manner to keep the device or intervention running smoothly. This requires a fair amount of technology literacy not only on the part of end users but also on the study staff assisting research participants to ensure adequate assistance with troubleshooting when problems arise. Regardless of the amount of advanced planning and testing, unanticipated issues with the technology will arise with any intervention, and these will affect recipients and study staff. Consequently, interventionists need to have a mechanism for receiving reports of technical problems or difficulties. Troubleshooting problems, assisting recipients, and communicating with developers require additional effort and expertise from interventionists. Given that the interventionists perform less of the intervention delivery, their role therefore shifts more to technical training and support for recipients and ongoing communication with developers.

#### Evidence-Based In-Person Interactions Do Not Translate Directly to Digital Interactions

##### Overview

Our interviews revealed the extent to which interventionists approached the creation of digital interventions in similar ways as they had in the past with traditional methods and how they could run into challenges as a result. Although this is related to the conceptualization of the design of an intervention, which we addressed in an earlier section, we see it as a broader mindset and therefore discuss it separately. We highlight that moving analog processes and behavioral interventions to a digital world is not a 1:1 replication, nor should it be. That which is efficient and effective offline may not translate to the digital world in the same way, and the benefit of digitization means that there are potential efficiencies that can be captured with redesigned processes that are not possible in an analog world. This applies both to the front end of an intervention that is visible to research participants and also to the back-end databases and structures that run the interventions. For example, one can certainly take print materials and data collection forms and put them in a digital format as is, but this may not be the most efficient and effective content delivery solution. Moreover, given that many behavioral interventions are delivered in a face-to-face format with trained interventionists using techniques such as MI, the switch to digital platforms, which are often automated, cannot be a perfect replication. Interviewees from this study raised several concerns about the translation of in-person interventions and interactions to a digital world, including issues related to intervention design and intervention fidelity.

##### Intervention Design

Despite access and literacy variability among intervention recipients, one of the potential benefits valued by researchers is the ability to reach more people through the use of digital interventions at scale [18]; however, this extended reach of digital interventions comes with added complexity for planning and implementing interventions. In addition to time zone considerations, there may be other regional or cultural factors that affect how the intervention will be experienced by recipients across geographic areas. An early understanding of the potential differences between populations in different areas will enable effective design and planning. In addition, the need for tech-savviness on the part of intervention recipients introduces a level of complexity that may be off-putting for individuals less comfortable with technology. This increases the need to introduce experts in human-computer interaction and interaction design, who specialize in developing conceptual models through which users can intuitively interact with technology (eg, the desktop on a computer interface is derived from the metaphor of a physical desktop with files and folders placed upon it). In contrast to supporting intervention planning by outlining a range of functional requirements to consider [28], focusing on user-centered conceptual models can help interventionists pivot from how they think of traditional interventions to how they might conceptualize digital interventions differently. Although some interventions try to emulate human counselors in order to provide an approach that mirrors real life, models other than that of the human counselor should be explored to effectively guide recipients through an intervention and to conceptualize how digital interventions can complement in-person services. Previous work has shown that although intervention recipients can develop a bond with an app and display open communication using this medium, the nature of the interaction is different than that with health professionals, as apps are not as able to simulate truly human qualities such as friendliness and collaboration [29]. Indeed, simulating realistic exchanges between humans and computers within the context of health issues has the potential to feel to human users as disingenuous, contrived, or overly stereotypical, which may be a deterrent to use [30]. There is evidence that suggests SBIRT (Screening, Brief Intervention, and Referral to Treatment) interventions can be at least as good as face-to-face, clinician-delivered interventions [31,32].

Interviewees often talked about the loss of ability to gauge and react to intervention recipients’ responses, behaviors, and body language. The ability of automated interventions to handle such nuanced verbal and nonverbal communication is still limited; however, we note that artificial intelligence and other emerging technologies do have the potential to improve personalization and adaptivity of interventions through speech pattern recognition, natural language processing, and machine learning. These advanced technological methods can help to process the large number of possible intervention pathways and make decisions based on certain rules that are preprogrammed or learned over time, addressing the challenge articulated by some interviewees. These innovations are developing and improving rapidly and mark an important area of study at this time. Furthermore, as interventionists look to digital interventions to supplement human interaction and support, there is a critical need for continued investigation of the recipient experience. Evidence is needed to determine the extent to which technology can convey empathy and emotional support and, if so, through which features or functions specifically. Finally, it should also be noted that although technology clearly cannot do everything that a human interventionist can do, neither can a human interventionist do everything that technology can do, and those advantages should be leveraged freely, rather than only seeking to replicate human interaction. The literature in this area includes examples of technology-delivered interventions performing equally well as human-delivered interventions [31], with similar overall acceptability [33] and greater cost-effectiveness [34].

### Limitations

This study was limited, first, by our focus on the CIAS platform, given that this work was conducted within the context of CIAS redesign activities. Although the limitation on CIAS users may have potentially introduced selection and response bias, the issues raised by interviewees in this study were largely focused on considerations for developing and implementing digital behavioral interventions in general, and not on CIAS itself. Our participants described experiences across a range of behavioral interventions. We also contextualized our recommendations within the broader literature and best practices for software projects. Finally, our sample size was small; however, saturation was achieved, leaving us confident that adding additional participants to the sample would yield little new information to contribute.

### Conclusions

Researchers from within the health and social sciences who wish to develop and implement digital behavioral interventions describe a consistent set of challenges. Issues resulting from lack of cross-disciplinary understanding; variability in recipients’ technology access, infrastructure, and literacy; and translating face-to-face interventions into digital interactions are common across multidisciplinary teams in this space and speak to the need for better training and planning among research teams who wish to work with digital health. Finding the right expertise to include on multidisciplinary teams can help teams overcome these challenges and build off similar work that has been done, as opposed to reinventing the wheel with every new project.

## Abbreviations

CIAS: Computerized Intervention Authoring System
COREQ: Consolidated Criteria for Reporting Qualitative Studies
MI: motivational interviewing
SBIRT: Screening, Brief Intervention, and Referral to Treatment
